# Methylphenidate and atomoxetine prescribing trends in children in the Western Cape Province of South Africa, 2005–2013

**DOI:** 10.4102/hsag.v23i0.1084

**Published:** 2018-07-31

**Authors:** Liezl Joubert, Johanita R. Burger, Ilse Truter, Martie S. Lubbe, Marike Cockeran

**Affiliations:** 1 Medicine Usage in South Africa, Faculty of Health Sciences, North-West University, South Africa; 2 Drug Utilisation Research Unit, Faculty of Health Sciences, Nelson Mandela University, South Africa

## Abstract

**Background:**

There is paucity of data on methylphenidate and atomoxetine prescribing patterns in South African children.

**Aim:**

To describe the prescribing trends of these agents in children residing in the Western Cape Province.

**Setting:**

South African private health sector.

**Methods:**

Longitudinal drug utilisation study on medicine claims data from 2005–2013, focussing on the number of patients and prescriptions per patient.

**Results:**

The total number of patients increased by 29.5% from 2005 to 2013. The majority were boys (male:female ratio, 3.5:1), and between the ages of > 6 and ≤12 years in 2005 and >12 and ≤18 years in 2013. More than 75% of patients received methylphenidate or atomoxetine in the City of Cape Town Metropolitan municipality. Prescriptions for methylphenidate and atomoxetine increased by 45.5% overall from 2005 to 2013 (*p* < 0.001), with that for methylphenidate and atomoxetine increasing by 36.0% and 314.5%, respectively. The average number of annual methylphenidate prescriptions per patient increased from 3.96 ± 2.92 (95% CI, 3.69–4.23) in 2005 to 4.38 ± 2.85 (95% CI, 4.14–4.61) in 2013 (Cohen’s *d* = 0.14) and for atomoxetine from 2.58 ± 1.86 (95% CI, 1.80–3.37) in 2005 to 4.85 ± 3.66 (95% CI, 3.84–5.86) in 2013 (Cohen’s *d* = 0.62).

**Conclusion:**

Although the total number of patients and prescribing of methylphenidate and atomoxetine increased significantly from 2005 to 2013, a slight downward trend was observed in the mean number of prescriptions per patient per year from 2008 onwards. These prescribing patterns warrant further research.

## Introduction

Several recent studies on the prescribing patterns of psychotropic medicine have indicated an increase in the number of prescriptions of methylphenidate and atomoxetine in children and adolescents (Boland et al. 2015; Dalsgaard, Nielsen & Simonsen 2013; Garfield et al. 2015; Prosser & Reid 2009; Prosser, Reid & Lambert 2014; Schellack & Meyer 2012; Truter 2009; 2014; Shyu et al. 2016; Venter 2004; Zito et al. 2008b). Reasons for such prescribing, however, are largely unavailable.

Prescribing of medication is influenced by several factors, which can include, *inter alia*, characteristics of the patient and his or her family, the socio-demographic status of patients and access to health insurance, the professional and prescribing characteristics of prescribers, drug attributes, the time of drug approval and introduction to the market, the marketing efforts of pharmaceutical companies, interpersonal communication among doctors and government policies (Heins, Bruggers, van Dijk & Korevaar 2016; Lublóy 2014). Cutts and Tet (2003) furthermore demonstrated in their study on Australian medical practitioners that those in rural areas perceive their location to affect their prescribing in conjunction with the distance of the patient’s residence to and from the practice, a lack of diagnostic facilities in the vicinity, the patients’ expectation of receiving a prescription and the level of monitoring that a patient may need during therapy. It was also found that doctors in remote areas prefer to prescribe the latest drug available on the market as they may have fewer side effects and would possibly minimise monitoring of the patient. However, this observation seemed to diminish as the practice became more rural, possibly explained by lack of pharmaceutical representation and information in these areas (Cutts & Tet 2003).

By using medical aid administrator data for 355 998 patients for a 1-month period in 2004, Truter (2009) showed that the Western Cape Province had, compared to other provinces in South Africa, the highest percentage of claims for methylphenidate for children aged 18 years and younger. Methylphenidate and atomoxetine are registered for the treatment of attention deficit hyperactivity disorder (ADHD); methylphenidate is also registered for the treatment of narcolepsy. Because of a lack of safety and efficacy data in children, methylphenidate and atomoxetine should not be administered to children younger than 6 years of age (Rossiter 2014; Snyman 2014).

Our knowledge of the effect of geographic area on the use of methylphenidate and atomoxetine, particularly in the Western Cape Province, is limited. The aim of this study was thus to investigate the prescribing patterns of methylphenidate and atomoxetine in children and adolescents under the age of 18 years in the Western Cape Province by analysing medical claims data over a period of nine consecutive years.

## Method and materials

### Study design

This study followed a longitudinal design. A retrospective, descriptive, drug utilisation review was conducted by analysing medicine claims data over a 9-year period. These claims were provided by one of the largest pharmaceutical benefit management (PBM) companies for medical aid schemes in South Africa. Between 01 January 2005 and 31 December 2013, this database comprised of 56 442 797 prescription claims in total for the Western Cape Province.

The data fields available for this study included the following: The National Pharmaceutical Product Interface codes (NAPPI-codes), a treatment date, a patient’s dependant code, an encrypted clinician code, active substances dispensed (trade and generic names), the quantity of each substance supplied, the strength, treatment period, gender, date of birth, ICD-10 code and the postal code of the provider claiming each medicine item for reimbursement. Geographic location of patients (municipal main place and district) was determined using the provider’s location (postal code) as proxy.

### Study population

The study population comprised of all children under the age of 18 years on the database residing in the Western Cape Province, South Africa, receiving at least one prescription for methylphenidate or atomoxetine during the study period. Participants were categorised by gender and divided into three age groups: age group 1 (≤6 years), age group 2 (> 6 and ≤12 years) and age group 3 (> 12 and ≤18 years). The patients’ ages were calculated according to the age on the date of the claim in line with his or her date of birth taking the first of January of the following year as index date.

### Statistical analysis

**FIGURE 1:**
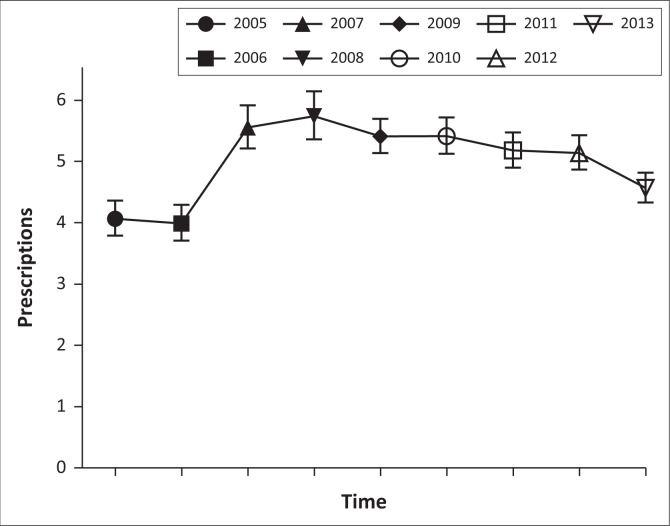
Change in the mean total number of prescriptions per patient per year from 2005 to 2013.

Drug utilisation metrics used in the study included the number of patients stratified by age, gender and district (Tables 1 and 2), and the total and average number of yearly prescriptions per patient, stratified by age and gender (Table 3 and Figure 1).

**TABLE 1:** Patients receiving methylphenidate and atomoxetine from 2005 to 2013 stratified by gender and age.

Variables	2005 *n* (%)	2006 *n* (%)	2007 *n* (%)	2008 *n* (%)	2009 *n* (%)	2010 *n* (%)	2011 *n* (%)	2012 *n* (%)	2013 *n* (%)
Number of patients ≤ 18 years in the Western Cape, *N*	30 926	32 300	20 661	15 269	25 142	23 466	21 262	20 795	23 456
Number of patients receiving methylphenidate and atomoxetine, *N* (%)^a^	448 (1.5)	497 (1.5)	363 (1.8)	345 (2.3)	563 (2.2)	549 (2.3)	491 (2.3)	528 (2.5)	580 (2.5)
**Gender, *n* (%)^b^**									
Boys	349 (77.9)	392 (78.9)	282 (77.7)	269 (78.0)	440 (78.2)	405 (73.8)	359 (73.1)	394 (74.6)	421 (72.6)
Girls	99 (22.1)	105 (21.1)	81 (22.3)	76 (22.0)	123 (21.9)	144 (26.2)	132 (26.9)	134 (25.4)	159 (27.4)
**Age (years), *n* (%)^b^**									
0 ≤ 6	1 (0.2)	2 (0.4)	3 (0.8)	1 (0.3)	1 (0.2)	5 (0.9)	4 (0.8)	1 (0.2)	7 (1.2)
> 6, ≤12	245 (54.7)	269 (54.1)	191 (52.6)	182 (52.8)	276 (49.0)	283 (51.6)	252 (51.3)	251 (47.5)	270 (46.6)
> 12, ≤18	202 (45.1)	226 (45.5)	169 (46.6)	162 (47.0)	286 (50.8)	261 (47.5)	235 (47.9)	276 (52.3)	303 (52.2)

a Percentages calculated using the total number of patients ≤ 18 years in the Western Cape Province, on the database (*N*) for each respective year, as denominator.

b Percentages calculated using the total number of patients ≤ 18 years in the Western Cape Province claiming methylphenidate and/or atomoxetine on the database (n) for each respective year, as denominator.

Data were analysed using Statistical Analysis System^®^ (SAS Institute Inc. 2002–2012). The number of patients in the study population and prescriptions claimed during the course of the study period were explained by means of descriptive statistics that included frequencies, means, standard deviations and 95% confidence intervals. Tests of association (chi-square test and Fishers’ exact test) were used to determine the association between totals of categorical data. The results were considered statistically significant if *p* ≤ 0.05. Cohen’s *d*-value was used to determine the effect size of the difference between the average number of prescriptions per patient by age group and gender with *d* ≥ 0.8 defined as a large effect with practical significance.

Changes in the annual number of methylphenidate and atomoxetine prescriptions per patient per year were modelled over time by fitting a repeated measures Poisson regression model using the generalised estimating equations procedure in SPSS IBM Corp. (2013). Pairwise comparisons were adjusted for multiple comparisons using the Bonferroni correction.

### Ethical considerations

The study was conducted according to the Declaration of Helsinki. Ethical approval was obtained from an authorised, licensed Research Ethics Committee before the commencement of the study (Ethics number: 00179-14-S1). Confidentiality was maintained at all times and data were analysed anonymously.

## Results and findings

Table 1 shows the demographic characteristics of the study population over the 9-year study period. The total number of children and adolescents under the age of 18 years receiving either methylphenidate or atomoxetine, as percentage of the total number of children and adolescents under the age of 18 years on the database in the Western Cape Province, ranged from 1.5% in 2005 to 2.5% in 2013. The number of patients identified per year fluctuated over the study period, however, increasing by 29.5% overall from 2005 to 2013. The majority were boys (male:female ratio 3.5:1 over the full study period) and were of age groups 2 (> 6 and ≤12 years) and 3 (> 12 and ≤18 years). Patients aged ≤6 years (age group 1) increased from 1 in 2005 to 7 in 2013, in comparison with a 10.2% increase in age group 2 (> 6 and ≤12 years) and 50.0% increase in age group 3 (> 12 and ≤18 years) (Table 1).

Table 2 depicts the distribution of patients receiving methylphenidate and atomoxetine over the study period, stratified by district and municipal main place. Prevalence by district and local municipality fluctuated over the study period. Overall, more than 75% of patients in the study population received methylphenidate or atomoxetine in the City of Cape Town Metropolitan Municipality. Prevalence in this municipality, however, increased by 20.5% from 2005 to 2013. Patients receiving methylphenidate or atomoxetine in the Cape Winelands district increased by 52.0% from 2005 to 2013, with prevalence in the Breede Valley and Drakenstein municipalities increasing by 200.0% and 92.3%, respectively. Prevalence in Stellenbosch decreased by 11.5% from 2005 to 2013. Patients receiving methylphenidate or atomoxetine in the Eden district increased from 26 in 2005 to 41 in 2013. In particular, the number of patients in Mossel Bay increased from four in 2005 to 13 in 2013. Patients receiving methylphenidate or atomoxetine in the Overberg and West Coast districts increased by 128.6% and 316.7%, respectively, during the study period. Prevalence in these local municipalities in particular increased most for Overstrand (from two cases in 2005 to 10 in 2013) and Saldanha (from four cases in 2005 to 14 in 2013).

**TABLE 2:** Patients receiving methylphenidate and atomoxetine from 2005 to 2013, stratified by district and municipality.

District or local municipality	2005 *n* (%)	2006 *n* (%)	2007 *n* (%)	2008 *n* (%)	2009 *n* (%)	2010 *n* (%)	2011 *n* (%)	2012 *n* (%)	2013 *n* (%)
Number of patients receiving methylphenidate and atomoxetine, *N* (%)^a^	460 (1.5)	525 (1.6)	379 (1.8)	365 (2.4)	596 (2.4)	570 (2.4)	505 (2.4)	548 (2.6)	605 (2.6)
**City of Cape Town Metropolitan (CCTM)**	371	417	280	260	445	448	390	400	447
Cape Winelands	50	58	56	48	87	62	60	77	76
Langeberg	1 (2.0)	2 (3.4)	-	1 (2.1)	2 (2.3)	-	-	2 (2.6)	1 (1.3)
Witzenberg	1 (2.0)	1 (1.7)	2 (3.6)	1 (2.1)	-	1 (1.6)	-	-	-
Breede Valley	9 (18.0)	11 (19.0)	11 (19.6)	10 (20.8)	18 (20.7)	16 (25.8)	15 (25.0)	22 (28.6)	27 (35.5)
Stellenbosch	26 (52.0)	34 (58.6)	28 (50.0)	27 (56.3)	39 (44.8)	24 (38.7)	23 (38.3)	22 (28.6)	23 (30.3)
Drakenstein	13 (26.0)	10 (17.2)	15 (26.8)	9 (18.8)	28 (32.2)	21 (33.9)	22 (36.7)	31 (40.3)	25 (32.9)
Central Karoo	-	1	-	-	2	-	-	-	
Prince Albert	-	-	-	-	-	-	-	-	-
Beaufort West	-	1 (100.0)	-	-	2 (100.0)	-	-	-	-
**Eden**	26	25	27	41	41	37	34	38	41
Mossel Bay	4 (15.4)	6 (24.0)	9 (33.3)	8 (19.5)	8 (19.5)	5 (13.5)	8 (23.5)	5 (13.2)	13 (31.7)
George	15 (57.7)	13 (52.0)	11 (40.7)	18 (43.9)	24 (58.5)	22 (59.5)	22 (64.7)	21 (55.3)	19 (46.3)
Knysna	2 (7.7)	3 (12.0)	3 (11.1)	3 (7.3)	4 (9.8)	4 (10.8)	1 (2.9)	8 (21.1)	5 (12.2)
Bitou	2 (7.7)	2 (8.0)	2 (7.4)	11 (26.8)	3 (7.3)	-	1 (2.9)	1 (2.6)	1 (2.4)
Oudtshoorn	3 (11.5)	1 (4.0)	2 (7.4)	1 (2.4)	1 (2.4)	5 (13.5)	2 (5.9)	3 (7.9)	2 (4.9)
Hessequa	-	-	-	-	1 (2.4)	1 (2.7)	-	-	1 (2.4)
**Overberg**	7	9	7	7	10	5	7	12	16
Swellendam	-	1 (11.1)	-	-	1 (1.0)	1 (20.0)	-	2 (16.7)	-
Theewaterskloof	5 (71.4)	2 (22.2)	3 (42.6)	1 (14.3)	-	2 (40.0)	4 (57.1)	4 (33.3)	3 (18.8)
Overstrand	2 (28.6)	5 (55.6)	4 (57.1)	5 (71.4)	6 (60.0)	2 (40.0)	3 (42.9)	6 (50.0)	10 (62.5)
Cape Agulhas	-	1 (11.1)	-	1 (14.26)	3 (30.00)	-	-	-	3 (18.8)
**West Coast**	6	15	9	9	11	18	14	21	25
Swartland	1 (16.7)	2 (13.3)	1 (11.1)	1 (11.1)	2 (18.2)	3 (16.7)	1 (7.1)	4 (19.1)	3 (12.0)
Bergrivier	1 (16.7)	4 (26.7)	1 (11.1)	1 (11.1)	1 (9.1)	4 (22.2)	4 (28.6)	5 (23.8)	1 (4.0)
Cederberg	-	-	1 (11.1)	1 (11.1)	-	2 (11.1)	2 (14.3)	6 (28.6)	5 (20.0)
Saldanha	4 (66.7)	5 (33.3)	4 (44.4)	2 (22.2)	5 (45.5)	8 (44.4)	5 (35.7)	4 (19.1)	14 (56.0)
Matzikama	-	4 (26.7)	2 (22.2)	4 (44.4)	3 (27.3)	1 (5.6)	2 (14.3)	2 (9.5)	2 (8.0)

a Percentages calculated using the total number of patients ≤18 years in the Western Cape Province, on the database (*N*) for each respective year, as denominator. The total number of patients stratified by geographic location may differ from the total number of unique patients in the study population (Table 1). Geographic location for patients was determined using the postal code of the provider (i.e. the municipal main place where the medicine was claimed) as proxy. Patients who made use of different service providers located in different municipalities/districts could, therefore be counted more than once.

Table 3 depicts the general prescribing patterns of methylphenidate and atomoxetine from 2005 to 2013. The total number of prescriptions increased by 45.5% from 2005 to 2013 (*p* < 0.001). From 2005 to 2013, prescriptions for methylphenidate increased by 36.0%, compared to a 314.5% for atomoxetine. The male:female ratio stayed relatively constant over the study period for methylphenidate prescriptions. Overall, prescribing of methylphenidate increased by 30.8% from 2005 to 2013 for boys versus 54.4% for girls. For prescriptions containing atomoxetine, the male:female ratio fluctuated over the study period, with an overall increase of 416.2% in the percentage of boys from 2005 to 2013 versus 164.0% for girls. Analysis by age group shows that prescriptions for methylphenidate increased from 1 in 14 children in age group 1, compared to a 14.7% increase for children in age group 2 (> 6 and ≤12 years) and a 63.5% increase for children in age group 3 (> 12 and ≤18 years). There were no prescriptions for atomoxetine for patients aged ≤6 years. Prescriptions for patients in age group 2 (> 6 and ≤12 years) increased from 38 in 2005 to 88 in 2013, whereas prescriptions for children in age group 3 (>12 and ≤18 years) increased from 24 in 2005 to 169 in 2013 (Table 3).

**TABLE 3:** Prescriptions for methylphenidate and atomoxetine from 2005 to 2013, stratified by gender and age.

Variables	2005 *n* (%)	2006 *n* (%)	2007 *n* (%)	2008 *n* (%)	2009 *n* (%)	2010 *n* (%)	2011 *n* (%)	2012 *n* (%)	2013 *n* (%)
**Number of methylphenidate and atomoxetine prescriptions in total population (database), *N* (%)**	1821	1982	2016	1980	3044	2973	2543	2714	2650
Total number of prescriptions for methylphenidate, *n* (%)^a^	1759 (96.6)	1687 (85.1)	1736 (86.1)	1722 (87.0)	2657 (87.3)	2621 (88.2)	2254 (88.6)	2425 (89.4)	2393 (90.3)
**Gender, *n* (%)^b^**									
Boys	1373 (78.1)	1387 (82.2)	1335 (76.9)	1330 (77.2)	2088 (78.6)	1990 (75.9)	1695 (75.2)	1896 (78.2)	1796 (75.1)
Girls	386 (21.9)	300 (17.8)	401 (20.7)	392 (22.8)	569 (21.4)	631 (24.1)	559 (24.8)	529 (21.8)	597 (25.0)
**Age (years), *n* (%)^b^**									
0 ≤ 6	1 (0.1)	4 (0.2)	9 (0.5)	1 (0.1)	1 (0.04)	29 (1.1)	9 (0.4)	2 (0.1)	14 (0.6)
>6, ≤ 12	1015 (57.7)	976 (57.9)	988 (56.9)	991 (57.6)	1376 (51.8)	1387 (52.9)	1243 (55.2)	1175 (48.5)	1164 (48.6)
>12, ≤ 18	743 (42.2)	707 (41.9)	739 (42.6)	730 (42.4)	1280 (48.2)	1205 (46.0)	1002 (44.5)	1248 (51.5)	1215 (50.8)
Average number of methylphenidate prescriptions per patient, mean ± SD (95% CI)	3.96 ± 2.92 (3.69–4.23)	3.80 ± 3.11 (3.51–4.09)	5.21 ± 3.14 (4.88–5.55)	5.45 ± 3.30 (5.08–5.82)	5.08 ± 3.10 (4.81–5.35)	5.06 ± 3.23 (4.78–5.34)	4.89 ± 3.08 (4.61–5.17)	4.83 ± 3.08 (4.56–5.10)	4.38 ± 2.85 (4.14–4.61)
Total number of prescriptions for atomoxetine, *n* (%)^a^	62 (3.5)	295 (17.5)	280 (16.1)	258 (15.0)	387 (14.6)	352 (13.4)	289 (12.8)	289 (11.9)	257 (10.7)
**Gender, *n* (%)^c^**									
Boys	37 (59.7)	224 (75.9)	238 (85.0)	195 (75.6)	291 (75.2)	229 (65.1)	213 (73.7)	172 (59.5)	191 (74.3)
Girls	25 (40.3)	71 (24.1)	42 (15.0)	63 (24.4)	96 (24.8)	123 (34.9)	76 (26.3)	117 (40.5)	66 (25.7)
**Age (years), *n* (%)^c^**									
0 ≤ 6	-	-	-	-	-	-	-	-	-
6, ≤ 12	38 (61.3)	192 (65.1)	123 (43.9)	182 (70.5)	215 (55.6)	178 (50.6)	148 (51.2)	105 (36.3)	88 (34.2)
> 12, ≤ 18	24 (38.7)	103 (34.9)	157 (56.1)	76 (29.5)	172 (44.4)	174 (49.4)	141 (48.8)	184 (63.7)	169 (65.8)
Average number of atomoxetine prescriptions per patient, mean ± SD (95% CI)	2.58 ± 1.86 (1.80–3.37)	3.78 ± 3.74 (2.94–4.63)	5.96 ± 4.15 (4.74–7.18)	6.45 ± 4.53 (5.00–7.90)	6.14 ± 3.81 (5.18–7.10)	6.77 ± 4.07 (5.64–7.90)	5.35 ± 3.74 (4.33–6.37)	5.67 ± 3.85 (4.59–6.75)	4.85 ± 3.66 (3.84–5.86)

a Percentages calculated using the total number of prescriptions on the database (*N*) for each respective year, as denominator.

b Percentages calculated using the total number of prescriptions for methylphenidate on the database (*n*) for each respective year, as denominator.

c Percentages calculated using the total number of prescriptions for atomoxetine (*n*) on the database for each respective year, as the denominator.

The mean number of methylphenidate prescriptions per patient per year increased marginally from 3.96 ± 2.92 (95% CI, 3.69–4.23) in 2005 to 4.38 ± 2.85 (95% CI, 4.14–4.61) in 2013 (Table 3). This difference in means was not practically significant (Cohen’s *d* = 0.14). The mean number of atomoxetine prescriptions per patient per year increased from 2.58 ± 1.86 (95% CI, 1.80–3.37) in 2005 to 4.85 ± 3.66 (95% CI, 3.84–5.86) in 2013. This difference was moderate (Cohen’s *d* = 0.62). Treating year as a fixed effect, it is shown that a significant change in the mean number of prescriptions per patient per year occurred over time (*c*^2^(8) = 123.457, *p* < 0.001). Based on Figure 1 (the estimated marginal means), there was a steep increase in the total mean number of prescriptions between 2006 and 2007, where after the incline seemed to lower (2007 to 2008), and then followed a slight downward trend towards 2013.

## Discussion

In the light of the paucity of data on the geographical prescribing patterns of methylphenidate and atomoxetine in children in South Africa, this longitudinal study aimed to describe these prescribing patterns in children under the age of 18 years in the Western Cape Province in particular, using medicine claims data obtained from a large PBM company. We identified a number of key findings.

We determined that approximately 1.5% – 2.5% of the total number of children and adolescents in the Western Cape Province on the database received either methylphenidate or atomoxetine over the 9-year study period. Prevalence by age, gender, district and local municipality fluctuated over the study period; however, it was evident that the majority of patients in the study population received methylphenidate and atomoxetine in the City of Cape Town Metropolitan Municipality. Geographic variations in the treatment of children with ADHD and anxiety in particular may be ascribed to differences in the understanding and belief of the role of pharmacotherapy among clinicians, and the availability of local expertise (Lui et al. 2014). According to Jack et al. (2014), there is a substantial mental health workforce shortage in South Africa, with 1.2 psychiatrists and 7.5 psychiatric nurses per 100 000 people, the majority of whom practise in urban locations. The City of Cape Town Metropolitan Municipality constitutes 64.2% of the total population in the Western Cape Province (as per our calculation based on the figures obtained from the Local Government Handbook for South Africa) (Main 2015). Approximately 15% of the population who are beneficiaries of medical aid schemes furthermore resides in the Western Cape Province along with 17.3% of the country’s general practitioners (15 general practitioners per 10 000 people in the private sector) (Council for Medical Schemes 2015), which could explain the higher prevalence observed in the City of Cape Town Metropolitan Municipality. The fluctuations observed may be ascribed to variations in the patient mix on the database per study year, and also to patient claim behaviour.

Analysis by age group showed that the highest prevalence of methylphenidate and atomoxetine prescribing was in the age group 2 (> 6 and ≤12 years) in 2003 and in the age group 3 (> 12 and ≤18 years) in 2013, with prevalence in the latter group increasing by 50% from 2005 to 2013. These findings agree with several other reports, for example the studies by Garfield et al. (2015), Schellack and Meyer (2012) and Venter (2004). Our lack of information on indication for prescribing, however, limits the evaluation of these findings. One of the registered uses of methylphenidate and atomoxetine – ADHD – is among the most prevalent mental illnesses of modern times (National Institute of Mental Health 2012), generally affecting between 2% and 5% of school-aged children and adolescents in the United Kingdom (UK) (National Health Service [NHS] 2014), compared to 11.0% in the United States (Centers for Disease Control 2014), and approximately 8% – 10% in South Africa (Attention Deficit and Hyperactivity Support Group of South Africa 2015). Narcolepsy, on the contrary, is a chronic disease commonly diagnosed in middle adulthood, with first symptoms often appearing in childhood or adolescence (Babiker & Prasad 2015:557). The exact prevalence of narcolepsy in the paediatric population is not known (Nevsimalova 2014; Peacock & Benca 2010); still, the condition is not considered rare. For example, prevalence of narcolepsy in European countries varies from 0.02% to 0.05% (Doherty, Crowe & Sweeney 2010; Ohayon et al. 2002). Compared to these statistics, the number of patients from our study population receiving treatment was relatively low. It should be taken into account that our study population represented only those patients with medical aid benefits from one data source, in one province of the country. A small number of children below the age of 6 years also received prescriptions for methylphenidate during the study period. Although the use of methylphenidate in children under the age of 6 years is not recommended, it is an accepted practice as these medications are not routinely tested in young children; however, close clinical monitoring of children receiving these drugs is warranted until clinical trials can establish the efficacy and safety thereof (Rossiter 2014; Zito et al. 2008b).

The majority of patients in our study population were boys (with a ratio of three to four male diagnoses for every female diagnosis), which is consistent with the findings from other South African studies conducted on patients with ADHD or receiving treatment with methylphenidate and atomoxetine (Schellack & Meyer 2012; Truter 2005; 2009; 2014; Venter 2004) as well as to international studies (American Psychiatric Association 2013; NHS 2014; Skogli et al. 2013). According to Schellack and Meyer (2012), the type of ADHD may influence gender-based statistics. This was shown by Van der Westhuizen (2010) determining the ratio of male to female to be four male diagnoses for every female diagnosis for predominantly hyperactive type ADHD diagnosis, and the gender ratio for the primarily inattentive type of ADHD was two male diagnoses for every female diagnosis. Skogli et al. (2013) further noted in their study that a higher risk of behavioural problems in boys with ADHD symptoms may be one reason for the referral of more boys than girls for clinical evaluation of ADHD. Narcolepsy, on the contrary, affects an equal number of boys and girls (Mindell & Owens 2010), although in adults, the prevalence appears to be higher in men than in women (Longstreth et al. 2007).

There was a statistically significant increase in the total number of methylphenidate and atomoxetine prescriptions claimed overall from 2005 to 2013. These trends are in line with international studies. For example Olfson et al. (2010) conducted a study on the trends in antipsychotic medication use in young children and have found that the rate at which these drugs were used approximately doubled from 1999 to 2001 and 2007. These prescriptions were inclusive of children receiving treatment for ADHD where it was found that there was a corresponding increase in the use of psychotropic medication for these children over the study period. Possible reasons for these findings include the treatment of other psychiatric conditions diagnosed along with ADHD (e.g. bipolar mood disorder) (Hassan, Agha & Thapar 2011). Most prescriptions in our study were for methylphenidate; however, prescriptions for atomoxetine increased proportionally more than that of methylphenidate over the 9-year study period. This trend was also shown by previous studies conducted in South Africa (Truter 2005; 2014) as well as international studies (Boland et al. 2015; Shyu et al. 2016). Based on a recent meta-analysis on the comparative efficacy and acceptability of immediate-release methylphenidate and atomoxetine in the treatment of ADHD in children and adolescents, the drugs have comparable efficacy and equal acceptability (Hanwella, Senanayake & de Silva 2011). Osmotically released methylphenidate was, however, considered more effective than atomoxetine in the treatment of ADHD in children and adolescents and should therefore be regarded as first-line therapy or the gold standard (Hanwella et al. 2011). If we presume that the majority of prescriptions in our study was in fact used for the treatment of AHDH, it may possibly explain the majority of methylphenidate prescriptions in our study. Compared to methylphenidate, atomoxetine is not a stimulant and thus not dependence forming (Gibson et al. 2006). Atomoxetine was registered with the South African Medicines Control Council in June 2005 (Fivas 2006); the release of the new product and marketing initiatives could explain the rather large increase (~4%) in use from 2005 to 2006, whereas overall increase in use towards 2013 could possibly be because of general acceptance of the product and proven effectiveness, with less side effects such as insomnia (Graham & Coghill 2008).

From the analysis of the changes in the annual number of methylphenidate and atomoxetine prescriptions per patient per year, it appeared as if the mean number of prescriptions per patient per year increased from 2006 to 2007 and then followed a slight downward trend. Possible reasons could include shorter treatment periods or the presence of drug holidays resulting in patients claiming fewer prescriptions per year. The cost of medicine as well as the patient’s medical benefit package could also influence claim patterns. Further investigation using a longer study period and larger sample of data including other provinces is, however, warranted to confirm these speculations.

## Conclusion and limitations

The findings of this longitudinal study indicate that although the total number of patients and prescribing of methylphenidate and atomoxetine in the Western Cape Province increased significantly from 2005 to 2013, a slight downward trend was observed in the mean number of prescriptions per patient per year from 2008 onwards. These prescribing patterns warrant further research. Prescribing of methylphenidate to children under the age of 6 years should also be accessed in terms of appropriateness.

The limitations of this study included that data were obtained from only one of the PMBs in South Africa – generalisability and external validation of the data are therefore limited. Also data for the study included only claims that were actually reimbursed by the patient’s medical aid scheme. The results of this study may therefore be an underreporting of methylphenidate and atomoxetine prescribing prevalence among patients in the Western Cape Province on the database. Furthermore, although this study provides evidence of the use of methylphenidate and atomoxetine in children and adolescents in the Western Cape Province, it does not provide definitive evidence that these drugs are used for ADHD, narcolepsy or off-label indications that are being diagnosed among these children.
